# Bis(9-amino­acridinium) bis­(pyridine-2,6-dicarboxyl­ato)cuprate(II) trihydrate

**DOI:** 10.1107/S160053681003059X

**Published:** 2010-09-30

**Authors:** Zohreh Derikvand, Jafar Attar Gharamaleki, Helen Stoeckli-Evans

**Affiliations:** a Department of Chemistry, Faculty of Sciences, Islamic Azad University, Khorramabad Branch, Khorramabad, Iran; b Young Researchers Club, Islamic Azad University, North Tehran Branch, Tehran, Iran; c Institute of Physics, University of Neuchâtel, rue Emile-Argand 11, CH-2009 Neuchâtel, Switzerland

## Abstract

The asymmetric unit of the title compound, (C_13_H_11_N_2_)_2_[Cu(C_7_H_3_NO_4_)_2_]·3H_2_O, consists of one [Cu(pydc)_2_]^2−^ dianion (pydc is pyridine-2,6-dicarboxyl­ate), two 9-amino­acridinum monocations and three uncoordinated water mol­ecules. The Cu^II^ atom is coordinated by two pydc dianions acting as tridentate ligands, and forming five-membered chelate rings with copper(II) as the central atom. The Cu^II^ atom is surrounded by four O atoms in the equatorial plane and two pyridine N atoms in axial positions, resulting in a distorted octa­hedral coordination geometry. In the crystal, there are two types of O—H⋯O and N—H⋯O hydrogen-bonding synthons linking the anionic and cationic fragments and the water mol­ecules, namely *R*_4_^4^(16), and *R*_4_^2^(8). There are also weak C—H⋯O hydrogen bonds, π–π stacking inter­actions [the shortest centroid–centroid distance is 3.350 (2) Å], and a C—O⋯π inter­action [O⋯centroid distance = 3.564 (2) Å], which connect the various components into a three-dimensional network.

## Related literature

For complexes containing a copper(II) atom, pyridine-2,6-dicarboxlic acid and various bases, see: Yenikaya *et al.* (2009 ▶); Zafer Yeşilel *et al.* (2010 ▶); Du *et al.* (2006 ▶); Aghabozorg *et al.* (2006 ▶, 2009 ▶). For the crystal structure of (aacrH)_2_[Ni(pydc)_2_]·3H_2_O, (aacr = 9-amino­acridine), see: Derikvand & Olmstead (2010 ▶). For graph-set analysis, see: Bernstein *et al.* (1995 ▶). An independent determination of the title compound is reported in the following paper by Aghabozorg *et al.* (2010 ▶).
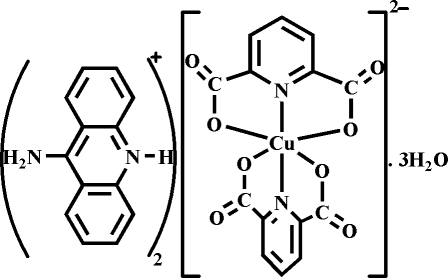


## Experimental

### 

#### Crystal data

(C_13_H_11_N_2_)_2_[Cu(C_7_H_3_NO_4_)_2_]·3H_2_O
*M* *_r_* = 838.27
Triclinic, 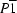

*a* = 10.8760 (16) Å
*b* = 13.283 (2) Å
*c* = 13.9820 (19) Å
α = 102.056 (12)°
β = 103.785 (11)°
γ = 105.573 (12)°
*V* = 1807.6 (5) Å^3^
*Z* = 2
Mo *K*α radiation
μ = 0.68 mm^−1^
*T* = 223 K
0.25 × 0.19 × 0.12 mm

#### Data collection

Stoe IPDS 2 diffractometer
Absorption correction: multi-scan (*MULscanABS*; Spek, 2009 ▶) *T*_min_ = 0.845, *T*_max_ = 0.920
19456 measured reflections
6819 independent reflections
4572 reflections with *I* > 2σ(*I*)
*R*_int_ = 0.060

#### Refinement

*R*[*F* ^2^ > 2σ(*F* ^2^)] = 0.038
*wR*(*F* ^2^) = 0.073
*S* = 0.87
6819 reflections
571 parameters
H atoms treated by a mixture of independent and constrained refinement
Δρ_max_ = 0.38 e Å^−3^
Δρ_min_ = −0.40 e Å^−3^

### 

Data collection: *X-AREA* (Stoe & Cie, 2006 ▶); cell refinement: *X-AREA*; data reduction: *X-RED32* (Stoe & Cie, 2006 ▶); program(s) used to solve structure: *SHELXS97* (Sheldrick, 2008 ▶); program(s) used to refine structure: *SHELXL97* (Sheldrick, 2008 ▶); molecular graphics: *PLATON* (Spek, 2009 ▶); software used to prepare material for publication: *SHELXL97* and *PLATON* (Spek, 2009 ▶).

## Supplementary Material

Crystal structure: contains datablocks I, global. DOI: 10.1107/S160053681003059X/vm2038sup1.cif

Structure factors: contains datablocks I. DOI: 10.1107/S160053681003059X/vm2038Isup2.hkl

Additional supplementary materials:  crystallographic information; 3D view; checkCIF report

## Figures and Tables

**Table 1:** Hydrogen-bond geometry (Å, °)

*D*—H⋯*A*	*D*—H	H⋯*A*	*D*⋯*A*	*D*—H⋯*A*
N6—H40⋯O1*W*	0.93 (3)	2.04 (3)	2.927 (4)	159 (2)
O2*W*—H41⋯O6^i^	0.87 (4)	1.93 (4)	2.796 (3)	177 (4)
N4—H42⋯O4^ii^	0.86 (3)	1.97 (3)	2.808 (3)	165 (3)
N4—H43⋯O7^iii^	0.92 (4)	1.99 (4)	2.880 (3)	161 (3)
O3*W*—H44⋯O2*W*	0.86 (4)	1.86 (4)	2.720 (4)	176 (3)
N6—H45⋯O6	0.86 (3)	2.18 (3)	2.965 (3)	153 (3)
N5—H46⋯O8^iii^	0.82 (3)	1.91 (3)	2.719 (3)	173 (3)
O3*W*—H47⋯O4^iv^	0.84 (4)	1.95 (4)	2.780 (4)	168 (3)
O1*W*—H48⋯O5	0.87 (4)	1.97 (4)	2.828 (3)	174 (4)
N3—H49⋯O3*W*	0.84 (3)	1.86 (3)	2.698 (3)	170 (3)
O1*W*—H50⋯O2^v^	0.89 (5)	1.96 (5)	2.847 (4)	176 (4)
O2*W*—H51⋯O6	0.86 (6)	1.97 (5)	2.812 (4)	167 (4)
C3—H3⋯O2*W*^v^	0.94	2.57	3.266 (4)	131
C10—H10⋯O3^vi^	0.94	2.51	3.152 (3)	126
C19—H19⋯O4^ii^	0.94	2.49	3.401 (3)	163
C23—H23⋯O7^iii^	0.94	2.52	3.262 (3)	136

Symmetry codes: (i) 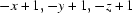; (ii) 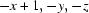; (iii) 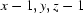; (iv) 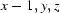; (v) 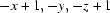; (vi) 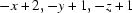.

**Fig. 1.**
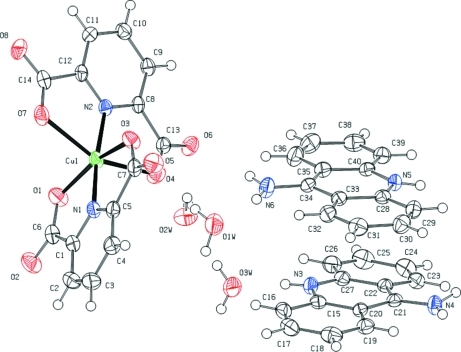
The molecular structure of the title compound with displacement ellipsoids drawn at the 50% probability level. H atoms are represented by spheres of arbitrary radius.

**Fig. 2.**
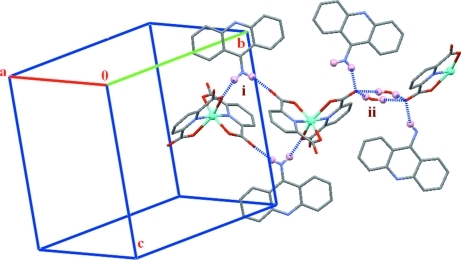
The one dimensional chain generated by the N—H···O and O—H···O hydrogen bonds (dashed lines) involving the anionic and cationic fragments and the water molecules of crystallization [graph-set i = R44(16), and graph-set ii = R24 (8)]. H atoms not involved in H bonding have been omitted for clarity.

**Fig. 3.**
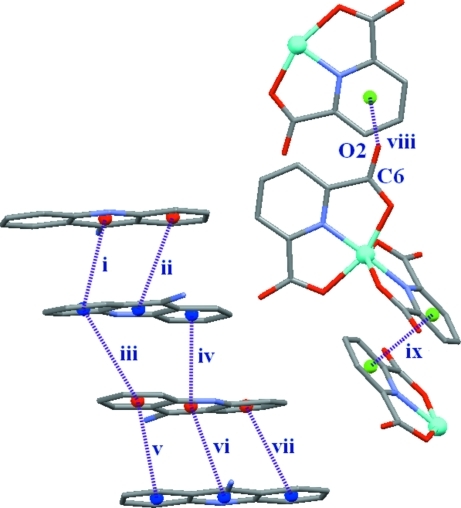
A view of the extensive π–π stacking interactions (dashed line) involving the aromatic rings of the 9-aminoacridinium ions, and the C–O···π interaction (viii, dashed line), involving the C6═O2 and the centroid of the pyridyl ring of a neighboring pydc ligand [Centroid-to-centroid distances: (i) 3.761 Å; (ii) 3.554 Å; (iii) 3.872 Å; (iv) 3.350 Å; (v) 3.668 Å; (vi) 3.842 Å; (vii) 3.834 Å; (ix) 3.768 Å]. H atoms have been omitted for clarity.

## supplementary crystallographic information

### Comment

A number of complexes containing a copper(II) atom, pyridine-2,6-dicarboxlic acid and various bases have been reported (Yenikaya *et al.*, 2009; Zafer Yeşilel *et al.,* 2010; Du *et al.,* 2006; Aghabozorg *et al.,* 2006, 2009). Herein, we report on the crystal structure of the title compound, that consists of a discrete [Cu(pydc)_2_]^2-^ dianion, two 9-aminoacridinum monocations and three uncoordinated water molecules (Fig. 1).

The copper(II) atom is coordinated by two pyridine-2,6-dicarboxylate anions (pydc) acting as tridentate ligands, forming five membered chelate rings. The metal center is surrounded by four oxygen atoms (O1, O3, O5 and O7) in the equatorial plane and by two pyridine nitrogen atoms (N1 and N2) in axial positions. In the anionic complex the N1—Cu1—N2 angle of 174.13 (8)° deviates significantly from linearity. The coordination geometry around the copper(II) atom is distorted octahedral (CuN_2_O_4_), and the valence angles vary considerabley from the required 90° and 180° in the basal plane *i.e.* 75.13 (8) - 159.50 (8) °. The (pydc)^2–^ ligands are almost orthogonal, with a dihedral angle involving the pyridine ring mean planes of 83.78 (13)°.

In the crystal two types of O–H···O and N–H···O hydrogen bond synthons are found namely, i [*R*^4^_4 _(16)], and ii [*R*^2^_4_(8)] (Bernstein *et al.*, 1995) [Table 1]. As shown in Fig. 2 they link the anionic and cationic fragments and the lattice water molecules to form a chain propagating in (110). Other intermolecular interactions are also present and include weak C–H···O hydrogen bonds, π–π stacking interactions [i-vii,ix in Fig. 3; the shortest centroid-to-centroid distance is 3.350 (2) Å], and a C–O···π interaction [viii in Fig. 3; O···centroid distance = 3.564 (2) Å], as shown in Fig. 3.

The crystal structure of the title compound is similar to that of (aacH)_2_[Ni(pydc)_2_]^.^ 3H_2_O, (aacr = 9-aminoacridine) (Derikvand *et al.* 2010).

### Experimental

An aqueous solution of copper(II) nitrate hexahydrate (0.5 mmol, 145 mg) in distilled water (5 ml) was added to a methanolic solution of pyridine-2,6-dicarboxylic acid (1 mmol, 167 mg) in distilled water (20 ml) and 9-aminoacridine (1 mmol, 194 mg) in methanol (5 ml) under stirring at 353 K, in a 1:2:2 molar ratio. The pale-green precipitate produced was dissolved in H_2_O/DMSO with the volume ratio of 1:4 (2/8 ml). Green plate-like crystals, suitable for X-ray characterization, were obtained after 3 days at room temperature.

### Refinement

The NH, NH_2_ and water H-atoms were located in difference Fourier maps and were refined freely: N—H = 0.82 (3) - 0.93 (3) Å, O—H = 0.84 (4) - 0.89 (5) Å. The C-bound H-atoms were included in calculated positions and treated as riding atoms: C—H = 0.94 Å with *U*_iso_(H) = 1.2*U*_eq_(parent C-atom).

### Crystal data

**Table:**

(C_13_H_11_N_2_)_2_[Cu(C_7_H_3_NO_4_)_2_]·3H_2_O	*Z* = 2
*M**_r_* = 838.27	*F*(000) = 866
Triclinic, *P*1	*D*_x_ = 1.540 Mg m^−^^3^
Hall symbol: -P 1	Mo *K*α radiation, λ = 0.71073 Å
*a* = 10.8760 (16) Å	Cell parameters from 10347 reflections
*b* = 13.283 (2) Å	θ = 1.6–26.1°
*c* = 13.9820 (19) Å	µ = 0.68 mm^−^^1^
α = 102.056 (12)°	*T* = 223 K
β = 103.785 (11)°	Plate, green
γ = 105.573 (12)°	0.25 × 0.19 × 0.12 mm
*V* = 1807.6 (5) Å^3^	

### Data collection

**Table:**

Stoe IPDS 2 diffractometer	6819 independent reflections
Radiation source: fine-focus sealed tube	4572 reflections with *I* > 2σ(*I*)
graphite	*R*_int_ = 0.060
φ + ω scans	θ_max_ = 25.8°, θ_min_ = 1.6°
Absorption correction: multi-scan (MULscanABS; Spek, 2009)	*h* = −13→13
*T*_min_ = 0.845, *T*_max_ = 0.920	*k* = −16→15
19456 measured reflections	*l* = −17→17

### Refinement

**Table:**

Refinement on *F*^2^	Primary atom site location: structure-invariant direct methods
Least-squares matrix: full	Secondary atom site location: difference Fourier map
*R*[*F*^2^ > 2σ(*F*^2^)] = 0.038	Hydrogen site location: inferred from neighbouring sites
*wR*(*F*^2^) = 0.073	H atoms treated by a mixture of independent and constrained refinement
*S* = 0.87	*w* = 1/[σ^2^(*F*_o_^2^) + (0.0307*P*)^2^] where *P* = (*F*_o_^2^ + 2*F*_c_^2^)/3
6819 reflections	(Δ/σ)_max_ = 0.001
571 parameters	Δρ_max_ = 0.38 e Å^−^^3^
0 restraints	Δρ_min_ = −0.40 e Å^−^^3^

### Special details

**Table:**

Geometry. Bond distances, angles *etc*. have been calculated using the rounded fractional coordinates. All su's are estimated from the variances of the (full) variance-covariance matrix. The cell e.s.d.'s are taken into account in the estimation of distances, angles and torsion angles
Refinement. Refinement of *F*^2^ against ALL reflections. The weighted *R*-factor *wR* and goodness of fit *S* are based on *F*^2^, conventional *R*-factors *R* are based on *F*, with *F* set to zero for negative *F*^2^. The threshold expression of *F*^2^ > σ(*F*^2^) is used only for calculating *R*-factors(gt) *etc*. and is not relevant to the choice of reflections for refinement. *R*-factors based on *F*^2^ are statistically about twice as large as those based on *F*, and *R*- factors based on ALL data will be even larger.

### Fractional atomic coordinates and isotropic or equivalent isotropic displacement parameters (Å^2^)

**Table:**

	*x*	*y*	*z*	*U*_iso_*/*U*_eq_	
Cu1	0.85975 (4)	0.23585 (3)	0.58073 (2)	0.0256 (1)	
O1	0.7445 (2)	0.20489 (15)	0.67478 (13)	0.0360 (6)	
O2	0.5978 (2)	0.06732 (17)	0.70104 (14)	0.0433 (7)	
O3	0.95569 (19)	0.20720 (14)	0.47238 (12)	0.0323 (6)	
O4	0.9737 (2)	0.07042 (15)	0.35947 (13)	0.0382 (6)	
O5	0.69468 (19)	0.28229 (14)	0.47403 (13)	0.0367 (6)	
O6	0.64662 (19)	0.43248 (15)	0.45922 (13)	0.0337 (6)	
O7	1.06223 (19)	0.28284 (15)	0.70913 (12)	0.0332 (6)	
O8	1.2315 (2)	0.43696 (17)	0.80731 (14)	0.0464 (7)	
N1	0.7888 (2)	0.08032 (17)	0.53178 (14)	0.0257 (7)	
N2	0.9302 (2)	0.39729 (16)	0.61666 (14)	0.0223 (6)	
C1	0.6994 (3)	0.0270 (2)	0.57121 (18)	0.0276 (8)	
C2	0.6431 (3)	−0.0846 (2)	0.5344 (2)	0.0363 (9)	
C3	0.6827 (3)	−0.1405 (2)	0.4588 (2)	0.0392 (10)	
C4	0.7773 (3)	−0.0835 (2)	0.42055 (19)	0.0323 (9)	
C5	0.8276 (3)	0.0285 (2)	0.45818 (17)	0.0262 (8)	
C6	0.6768 (3)	0.1051 (2)	0.65601 (18)	0.0312 (9)	
C7	0.9271 (3)	0.1077 (2)	0.42669 (17)	0.0271 (8)	
C8	0.8614 (2)	0.45192 (19)	0.56787 (16)	0.0210 (7)	
C9	0.9172 (3)	0.5622 (2)	0.58274 (18)	0.0258 (8)	
C10	1.0461 (3)	0.6180 (2)	0.64911 (18)	0.0286 (9)	
C11	1.1150 (3)	0.5620 (2)	0.70013 (18)	0.0269 (8)	
C12	1.0549 (3)	0.4513 (2)	0.68252 (17)	0.0237 (8)	
C13	0.7221 (3)	0.3827 (2)	0.49439 (17)	0.0253 (8)	
C14	1.1228 (3)	0.3841 (2)	0.73790 (18)	0.0282 (9)	
N3	0.1654 (2)	0.17225 (18)	0.11602 (17)	0.0297 (8)	
N4	0.0470 (2)	0.1067 (2)	−0.19910 (16)	0.0295 (8)	
C15	0.1780 (3)	0.0814 (2)	0.05922 (18)	0.0270 (8)	
C16	0.2329 (3)	0.0150 (2)	0.1093 (2)	0.0325 (9)	
C17	0.2452 (3)	−0.0764 (2)	0.0543 (2)	0.0382 (10)	
C18	0.2056 (3)	−0.1044 (2)	−0.0542 (2)	0.0360 (9)	
C19	0.1536 (3)	−0.0401 (2)	−0.1042 (2)	0.0292 (8)	
C20	0.1369 (2)	0.0542 (2)	−0.05001 (17)	0.0233 (8)	
C21	0.0824 (2)	0.1246 (2)	−0.09795 (17)	0.0240 (8)	
C22	0.0633 (2)	0.2156 (2)	−0.03506 (17)	0.0232 (8)	
C23	−0.0057 (3)	0.2806 (2)	−0.07617 (19)	0.0283 (8)	
C24	−0.0252 (3)	0.3638 (2)	−0.0140 (2)	0.0355 (10)	
C25	0.0242 (3)	0.3871 (2)	0.0935 (2)	0.0400 (10)	
C26	0.0888 (3)	0.3254 (2)	0.1365 (2)	0.0346 (9)	
C27	0.1076 (3)	0.2375 (2)	0.07306 (18)	0.0271 (8)	
N5	0.3572 (2)	0.39380 (17)	−0.02097 (16)	0.0264 (7)	
N6	0.5174 (3)	0.3113 (2)	0.23769 (18)	0.0355 (8)	
C28	0.3934 (2)	0.3036 (2)	−0.03271 (18)	0.0245 (8)	
C29	0.3761 (3)	0.2399 (2)	−0.13218 (18)	0.0299 (9)	
C30	0.4114 (3)	0.1489 (2)	−0.1451 (2)	0.0343 (9)	
C31	0.4661 (3)	0.1165 (2)	−0.0593 (2)	0.0334 (9)	
C32	0.4863 (3)	0.1782 (2)	0.03764 (19)	0.0302 (9)	
C33	0.4506 (2)	0.2735 (2)	0.05425 (18)	0.0252 (8)	
C34	0.4677 (3)	0.3401 (2)	0.15506 (18)	0.0266 (8)	
C35	0.4295 (3)	0.4357 (2)	0.16326 (19)	0.0291 (8)	
C36	0.4400 (3)	0.5062 (2)	0.2583 (2)	0.0382 (10)	
C37	0.3979 (3)	0.5932 (2)	0.2626 (2)	0.0414 (10)	
C38	0.3422 (3)	0.6171 (2)	0.1720 (2)	0.0375 (10)	
C39	0.3303 (3)	0.5512 (2)	0.0784 (2)	0.0304 (9)	
C40	0.3721 (3)	0.4599 (2)	0.07257 (17)	0.0247 (8)	
O1W	0.5825 (3)	0.1218 (2)	0.28006 (17)	0.0486 (8)	
O2W	0.3854 (3)	0.36532 (18)	0.47401 (17)	0.0440 (8)	
O3W	0.1873 (3)	0.2025 (2)	0.31711 (16)	0.0540 (9)	
H2	0.57860	−0.12260	0.56030	0.0440*	
H3	0.64550	−0.21700	0.43320	0.0470*	
H4	0.80620	−0.12060	0.37010	0.0390*	
H9	0.86790	0.59940	0.54800	0.0310*	
H10	1.08600	0.69310	0.65920	0.0340*	
H11	1.20220	0.59870	0.74650	0.0320*	
H16	0.26130	0.03420	0.18160	0.0390*	
H17	0.28020	−0.12140	0.08840	0.0460*	
H18	0.21510	−0.16760	−0.09210	0.0430*	
H19	0.12840	−0.05930	−0.17650	0.0350*	
H23	−0.03870	0.26600	−0.14800	0.0340*	
H24	−0.07190	0.40600	−0.04270	0.0430*	
H25	0.01250	0.44620	0.13620	0.0480*	
H26	0.12080	0.34130	0.20850	0.0420*	
H42	0.054 (3)	0.053 (2)	−0.241 (2)	0.039 (8)*	
H43	0.033 (3)	0.162 (3)	−0.226 (2)	0.052 (9)*	
H49	0.182 (3)	0.182 (2)	0.180 (2)	0.048 (9)*	
H29	0.33980	0.26070	−0.18990	0.0360*	
H30	0.39940	0.10690	−0.21180	0.0410*	
H31	0.48860	0.05240	−0.06910	0.0400*	
H32	0.52470	0.15690	0.09440	0.0360*	
H36	0.47720	0.49190	0.31980	0.0460*	
H37	0.40590	0.63840	0.32690	0.0500*	
H38	0.31330	0.67810	0.17570	0.0450*	
H39	0.29380	0.56730	0.01780	0.0360*	
H40	0.538 (3)	0.247 (2)	0.2337 (19)	0.031 (7)*	
H45	0.536 (3)	0.358 (3)	0.296 (2)	0.047 (9)*	
H46	0.317 (3)	0.401 (2)	−0.075 (2)	0.040 (9)*	
H48	0.620 (4)	0.168 (3)	0.341 (3)	0.075 (13)*	
H50	0.525 (5)	0.062 (4)	0.283 (3)	0.095 (16)*	
H41	0.372 (4)	0.427 (3)	0.494 (3)	0.064 (12)*	
H51	0.463 (5)	0.375 (3)	0.465 (3)	0.085 (15)*	
H44	0.247 (4)	0.254 (3)	0.368 (3)	0.076 (13)*	
H47	0.126 (4)	0.170 (3)	0.338 (2)	0.052 (11)*	

### Atomic displacement parameters (Å^2^)

**Table:**

	*U*^11^	*U*^22^	*U*^33^	*U*^12^	*U*^13^	*U*^23^
Cu1	0.0360 (2)	0.0211 (2)	0.0214 (2)	0.0101 (2)	0.0108 (1)	0.0067 (1)
O1	0.0498 (13)	0.0265 (10)	0.0348 (10)	0.0111 (10)	0.0216 (9)	0.0079 (8)
O2	0.0493 (14)	0.0480 (13)	0.0409 (11)	0.0142 (11)	0.0268 (11)	0.0181 (10)
O3	0.0482 (13)	0.0225 (10)	0.0300 (9)	0.0115 (9)	0.0195 (9)	0.0077 (8)
O4	0.0485 (13)	0.0350 (11)	0.0304 (9)	0.0109 (10)	0.0210 (9)	0.0023 (8)
O5	0.0386 (12)	0.0245 (11)	0.0364 (10)	0.0078 (9)	−0.0011 (9)	0.0054 (8)
O6	0.0309 (11)	0.0356 (11)	0.0324 (9)	0.0162 (9)	0.0014 (8)	0.0087 (8)
O7	0.0445 (12)	0.0312 (11)	0.0288 (9)	0.0182 (10)	0.0083 (9)	0.0151 (8)
O8	0.0397 (13)	0.0500 (13)	0.0382 (11)	0.0093 (11)	−0.0084 (10)	0.0211 (10)
N1	0.0330 (14)	0.0251 (12)	0.0204 (10)	0.0107 (10)	0.0074 (10)	0.0092 (9)
N2	0.0285 (13)	0.0231 (11)	0.0173 (9)	0.0108 (10)	0.0084 (9)	0.0056 (8)
C1	0.0307 (16)	0.0282 (14)	0.0244 (12)	0.0087 (12)	0.0067 (12)	0.0122 (11)
C2	0.0424 (19)	0.0287 (15)	0.0351 (15)	0.0047 (13)	0.0123 (14)	0.0130 (12)
C3	0.052 (2)	0.0208 (14)	0.0337 (15)	0.0023 (14)	0.0080 (14)	0.0046 (12)
C4	0.0439 (18)	0.0249 (14)	0.0244 (13)	0.0108 (13)	0.0087 (12)	0.0026 (11)
C5	0.0333 (16)	0.0249 (14)	0.0194 (12)	0.0113 (12)	0.0054 (11)	0.0059 (10)
C6	0.0363 (17)	0.0367 (16)	0.0246 (13)	0.0147 (14)	0.0105 (12)	0.0130 (12)
C7	0.0362 (17)	0.0272 (14)	0.0190 (12)	0.0121 (13)	0.0077 (11)	0.0082 (10)
C8	0.0255 (14)	0.0253 (13)	0.0166 (11)	0.0130 (11)	0.0086 (10)	0.0068 (10)
C9	0.0316 (16)	0.0260 (14)	0.0254 (12)	0.0152 (12)	0.0091 (12)	0.0116 (11)
C10	0.0358 (17)	0.0238 (14)	0.0272 (13)	0.0090 (12)	0.0110 (12)	0.0092 (11)
C11	0.0258 (15)	0.0265 (14)	0.0225 (12)	0.0045 (12)	0.0032 (11)	0.0056 (10)
C12	0.0278 (15)	0.0287 (14)	0.0162 (11)	0.0103 (12)	0.0085 (11)	0.0072 (10)
C13	0.0289 (15)	0.0268 (14)	0.0206 (12)	0.0099 (12)	0.0077 (11)	0.0069 (10)
C14	0.0355 (18)	0.0335 (16)	0.0244 (13)	0.0168 (14)	0.0132 (13)	0.0153 (12)
N3	0.0279 (14)	0.0378 (14)	0.0209 (11)	0.0070 (11)	0.0071 (10)	0.0095 (10)
N4	0.0405 (15)	0.0293 (13)	0.0200 (11)	0.0156 (12)	0.0081 (10)	0.0066 (10)
C15	0.0163 (14)	0.0335 (15)	0.0301 (13)	0.0035 (12)	0.0079 (11)	0.0128 (12)
C16	0.0237 (16)	0.0433 (17)	0.0334 (14)	0.0087 (13)	0.0092 (12)	0.0203 (13)
C17	0.0228 (16)	0.0460 (18)	0.0539 (18)	0.0124 (14)	0.0101 (14)	0.0325 (15)
C18	0.0275 (16)	0.0312 (16)	0.0531 (17)	0.0115 (13)	0.0147 (14)	0.0153 (13)
C19	0.0247 (15)	0.0277 (14)	0.0335 (14)	0.0077 (12)	0.0076 (12)	0.0089 (11)
C20	0.0168 (14)	0.0279 (14)	0.0233 (12)	0.0050 (11)	0.0049 (10)	0.0085 (10)
C21	0.0188 (14)	0.0257 (13)	0.0233 (12)	0.0024 (11)	0.0073 (11)	0.0045 (10)
C22	0.0202 (14)	0.0246 (13)	0.0229 (12)	0.0040 (11)	0.0081 (11)	0.0059 (10)
C23	0.0302 (16)	0.0267 (14)	0.0289 (13)	0.0090 (12)	0.0113 (12)	0.0081 (11)
C24	0.0381 (18)	0.0313 (16)	0.0433 (16)	0.0149 (14)	0.0195 (14)	0.0118 (13)
C25	0.050 (2)	0.0316 (16)	0.0405 (16)	0.0132 (15)	0.0256 (15)	0.0023 (13)
C26	0.0396 (18)	0.0324 (15)	0.0276 (13)	0.0051 (14)	0.0155 (13)	0.0035 (12)
C27	0.0228 (15)	0.0305 (14)	0.0257 (12)	0.0041 (12)	0.0094 (11)	0.0079 (11)
N5	0.0263 (13)	0.0301 (13)	0.0222 (11)	0.0090 (10)	0.0043 (10)	0.0107 (10)
N6	0.0403 (16)	0.0389 (15)	0.0252 (13)	0.0163 (13)	0.0029 (11)	0.0096 (12)
C28	0.0183 (14)	0.0267 (14)	0.0282 (12)	0.0060 (11)	0.0076 (11)	0.0092 (11)
C29	0.0273 (16)	0.0387 (16)	0.0246 (12)	0.0120 (13)	0.0078 (11)	0.0103 (11)
C30	0.0324 (17)	0.0392 (17)	0.0297 (14)	0.0126 (14)	0.0092 (12)	0.0065 (12)
C31	0.0280 (16)	0.0354 (16)	0.0407 (15)	0.0142 (13)	0.0128 (13)	0.0122 (13)
C32	0.0236 (15)	0.0363 (16)	0.0322 (14)	0.0113 (13)	0.0078 (12)	0.0126 (12)
C33	0.0171 (14)	0.0297 (14)	0.0282 (13)	0.0066 (12)	0.0053 (11)	0.0110 (11)
C34	0.0171 (14)	0.0314 (14)	0.0270 (13)	0.0037 (12)	0.0026 (11)	0.0107 (11)
C35	0.0236 (15)	0.0313 (15)	0.0293 (13)	0.0056 (12)	0.0070 (11)	0.0089 (11)
C36	0.0406 (19)	0.0424 (17)	0.0252 (13)	0.0116 (15)	0.0051 (13)	0.0059 (12)
C37	0.0445 (19)	0.0369 (17)	0.0322 (15)	0.0101 (15)	0.0075 (14)	−0.0017 (13)
C38	0.0342 (18)	0.0298 (15)	0.0470 (17)	0.0107 (13)	0.0126 (14)	0.0085 (13)
C39	0.0278 (16)	0.0296 (15)	0.0349 (14)	0.0104 (12)	0.0086 (12)	0.0122 (12)
C40	0.0192 (14)	0.0254 (13)	0.0253 (12)	0.0034 (11)	0.0052 (11)	0.0062 (11)
O1W	0.0624 (17)	0.0409 (14)	0.0332 (12)	0.0100 (13)	0.0065 (11)	0.0113 (11)
O2W	0.0368 (14)	0.0343 (13)	0.0539 (13)	0.0069 (11)	0.0122 (11)	0.0072 (10)
O3W	0.0522 (17)	0.0673 (17)	0.0235 (11)	−0.0061 (13)	0.0107 (11)	0.0096 (11)

### Geometric parameters (Å, °)

**Table:**

Cu1—O1	2.050 (2)	C3—H3	0.9400
Cu1—O3	2.063 (2)	C4—H4	0.9400
Cu1—O5	2.352 (2)	C9—H9	0.9400
Cu1—O7	2.3178 (19)	C10—H10	0.9400
Cu1—N1	1.906 (2)	C11—H11	0.9400
Cu1—N2	1.981 (2)	C15—C20	1.421 (3)
O1—C6	1.268 (3)	C15—C16	1.402 (4)
O2—C6	1.243 (4)	C16—C17	1.353 (4)
O3—C7	1.258 (3)	C17—C18	1.411 (4)
O4—C7	1.246 (3)	C18—C19	1.363 (4)
O5—C13	1.239 (3)	C19—C20	1.403 (4)
O6—C13	1.260 (4)	C20—C21	1.430 (4)
O7—C14	1.256 (3)	C21—C22	1.434 (3)
O8—C14	1.247 (3)	C22—C27	1.413 (3)
O1W—H48	0.87 (4)	C22—C23	1.410 (4)
O1W—H50	0.89 (5)	C23—C24	1.354 (4)
O2W—H41	0.87 (4)	C24—C25	1.405 (4)
O2W—H51	0.86 (6)	C25—C26	1.357 (4)
N1—C1	1.342 (4)	C26—C27	1.405 (4)
N1—C5	1.333 (3)	C16—H16	0.9400
N2—C12	1.345 (4)	C17—H17	0.9400
N2—C8	1.344 (3)	C18—H18	0.9400
O3W—H47	0.84 (4)	C19—H19	0.9400
O3W—H44	0.86 (4)	C23—H23	0.9400
N3—C27	1.356 (4)	C24—H24	0.9400
N3—C15	1.359 (4)	C25—H25	0.9400
N4—C21	1.325 (3)	C26—H26	0.9400
N3—H49	0.84 (3)	C28—C33	1.415 (3)
N4—H42	0.86 (3)	C28—C29	1.409 (3)
N4—H43	0.92 (4)	C29—C30	1.354 (4)
N5—C40	1.360 (3)	C30—C31	1.411 (4)
N5—C28	1.349 (3)	C31—C32	1.362 (4)
N6—C34	1.331 (4)	C32—C33	1.411 (4)
N5—H46	0.82 (3)	C33—C34	1.439 (3)
N6—H45	0.86 (3)	C34—C35	1.430 (4)
N6—H40	0.93 (3)	C35—C40	1.415 (4)
C1—C6	1.518 (4)	C35—C36	1.416 (4)
C1—C2	1.372 (4)	C36—C37	1.348 (4)
C2—C3	1.384 (4)	C37—C38	1.406 (4)
C3—C4	1.390 (4)	C38—C39	1.370 (4)
C4—C5	1.374 (4)	C39—C40	1.399 (4)
C5—C7	1.510 (4)	C29—H29	0.9400
C8—C13	1.521 (4)	C30—H30	0.9400
C8—C9	1.376 (4)	C31—H31	0.9400
C9—C10	1.382 (4)	C32—H32	0.9400
C10—C11	1.375 (4)	C36—H36	0.9400
C11—C12	1.381 (4)	C37—H37	0.9400
C12—C14	1.521 (4)	C38—H38	0.9400
C2—H2	0.9400	C39—H39	0.9400
			
O1—Cu1—O3	159.50 (8)	C12—C11—H11	120.00
O1—Cu1—O5	91.62 (8)	C10—C11—H11	120.00
O1—Cu1—O7	94.52 (7)	N3—C15—C20	120.4 (2)
O1—Cu1—N1	79.95 (9)	C16—C15—C20	120.2 (2)
O1—Cu1—N2	104.42 (8)	N3—C15—C16	119.4 (2)
O3—Cu1—O5	94.79 (7)	C15—C16—C17	120.5 (2)
O3—Cu1—O7	89.05 (7)	C16—C17—C18	120.1 (3)
O3—Cu1—N1	79.73 (8)	C17—C18—C19	120.2 (3)
O3—Cu1—N2	96.04 (8)	C18—C19—C20	121.3 (2)
O5—Cu1—O7	151.63 (7)	C15—C20—C21	118.4 (2)
O5—Cu1—N1	101.04 (8)	C19—C20—C21	124.0 (2)
O5—Cu1—N2	75.13 (8)	C15—C20—C19	117.6 (2)
O7—Cu1—N1	107.30 (8)	C20—C21—C22	119.1 (2)
O7—Cu1—N2	76.51 (8)	N4—C21—C22	119.2 (2)
N1—Cu1—N2	174.13 (8)	N4—C21—C20	121.7 (2)
Cu1—O1—C6	114.78 (18)	C21—C22—C23	123.0 (2)
Cu1—O3—C7	114.16 (18)	C23—C22—C27	118.0 (2)
Cu1—O5—C13	111.38 (17)	C21—C22—C27	118.9 (2)
Cu1—O7—C14	111.30 (18)	C22—C23—C24	121.1 (2)
H48—O1W—H50	110 (4)	C23—C24—C25	120.1 (3)
H41—O2W—H51	112 (4)	C24—C25—C26	120.8 (3)
Cu1—N1—C1	118.74 (17)	C25—C26—C27	119.8 (2)
Cu1—N1—C5	118.93 (19)	N3—C27—C26	119.6 (2)
C1—N1—C5	122.3 (2)	N3—C27—C22	120.2 (2)
Cu1—N2—C8	120.85 (16)	C22—C27—C26	120.1 (3)
C8—N2—C12	119.7 (2)	C17—C16—H16	120.00
Cu1—N2—C12	119.06 (18)	C15—C16—H16	120.00
H44—O3W—H47	109 (3)	C18—C17—H17	120.00
C15—N3—C27	122.8 (2)	C16—C17—H17	120.00
C27—N3—H49	118 (2)	C17—C18—H18	120.00
C15—N3—H49	118 (2)	C19—C18—H18	120.00
H42—N4—H43	116 (3)	C20—C19—H19	119.00
C21—N4—H43	119.1 (18)	C18—C19—H19	119.00
C21—N4—H42	123.8 (19)	C22—C23—H23	119.00
C28—N5—C40	123.1 (2)	C24—C23—H23	119.00
C28—N5—H46	114 (2)	C25—C24—H24	120.00
C40—N5—H46	123 (2)	C23—C24—H24	120.00
H40—N6—H45	121 (3)	C26—C25—H25	120.00
C34—N6—H45	116 (2)	C24—C25—H25	120.00
C34—N6—H40	123.0 (15)	C25—C26—H26	120.00
N1—C1—C2	119.8 (3)	C27—C26—H26	120.00
C2—C1—C6	128.8 (3)	N5—C28—C33	120.5 (2)
N1—C1—C6	111.5 (2)	C29—C28—C33	119.8 (2)
C1—C2—C3	119.1 (3)	N5—C28—C29	119.8 (2)
C2—C3—C4	120.0 (3)	C28—C29—C30	120.4 (2)
C3—C4—C5	118.4 (3)	C29—C30—C31	120.6 (2)
N1—C5—C7	111.5 (2)	C30—C31—C32	120.0 (3)
C4—C5—C7	128.1 (3)	C31—C32—C33	121.1 (2)
N1—C5—C4	120.3 (3)	C28—C33—C32	118.2 (2)
O1—C6—O2	126.2 (3)	C28—C33—C34	118.7 (2)
O2—C6—C1	118.9 (2)	C32—C33—C34	123.1 (2)
O1—C6—C1	114.9 (3)	N6—C34—C33	119.7 (3)
O3—C7—C5	115.7 (2)	N6—C34—C35	121.8 (2)
O4—C7—C5	118.5 (2)	C33—C34—C35	118.5 (2)
O3—C7—O4	125.8 (3)	C34—C35—C40	119.3 (2)
N2—C8—C13	115.3 (2)	C34—C35—C36	123.4 (2)
C9—C8—C13	123.6 (2)	C36—C35—C40	117.2 (3)
N2—C8—C9	121.1 (2)	C35—C36—C37	121.6 (3)
C8—C9—C10	119.5 (3)	C36—C37—C38	120.7 (3)
C9—C10—C11	119.0 (3)	C37—C38—C39	119.7 (3)
C10—C11—C12	119.3 (3)	C38—C39—C40	120.3 (2)
N2—C12—C11	121.3 (3)	N5—C40—C39	119.8 (2)
C11—C12—C14	122.6 (3)	C35—C40—C39	120.5 (2)
N2—C12—C14	116.1 (2)	N5—C40—C35	119.8 (3)
O5—C13—O6	126.4 (3)	C28—C29—H29	120.00
O5—C13—C8	116.4 (3)	C30—C29—H29	120.00
O6—C13—C8	117.2 (2)	C31—C30—H30	120.00
O7—C14—C12	116.3 (2)	C29—C30—H30	120.00
O8—C14—C12	115.6 (2)	C32—C31—H31	120.00
O7—C14—O8	128.1 (3)	C30—C31—H31	120.00
C1—C2—H2	120.00	C31—C32—H32	119.00
C3—C2—H2	120.00	C33—C32—H32	120.00
C2—C3—H3	120.00	C35—C36—H36	119.00
C4—C3—H3	120.00	C37—C36—H36	119.00
C3—C4—H4	121.00	C38—C37—H37	120.00
C5—C4—H4	121.00	C36—C37—H37	120.00
C8—C9—H9	120.00	C37—C38—H38	120.00
C10—C9—H9	120.00	C39—C38—H38	120.00
C9—C10—H10	120.00	C38—C39—H39	120.00
C11—C10—H10	121.00	C40—C39—H39	120.00
			
O3—Cu1—O1—C6	−11.1 (4)	N1—C5—C7—O3	−0.9 (4)
O5—Cu1—O1—C6	97.2 (2)	C4—C5—C7—O3	179.1 (3)
O7—Cu1—O1—C6	−110.5 (2)	C4—C5—C7—O4	−1.2 (5)
N1—Cu1—O1—C6	−3.7 (2)	N1—C5—C7—O4	178.9 (2)
N2—Cu1—O1—C6	172.3 (2)	C9—C8—C13—O6	−12.3 (4)
O1—Cu1—O3—C7	7.0 (3)	C13—C8—C9—C10	−178.3 (2)
O5—Cu1—O3—C7	−100.75 (19)	N2—C8—C13—O5	−11.4 (3)
O7—Cu1—O3—C7	107.40 (19)	N2—C8—C9—C10	−0.1 (4)
N1—Cu1—O3—C7	−0.37 (19)	C9—C8—C13—O5	166.8 (2)
N2—Cu1—O3—C7	−176.27 (19)	N2—C8—C13—O6	169.5 (2)
O1—Cu1—O5—C13	99.50 (19)	C8—C9—C10—C11	−1.0 (4)
O3—Cu1—O5—C13	−99.99 (19)	C9—C10—C11—C12	1.2 (4)
O7—Cu1—O5—C13	−3.1 (3)	C10—C11—C12—N2	−0.2 (4)
N1—Cu1—O5—C13	179.57 (19)	C10—C11—C12—C14	−178.4 (3)
N2—Cu1—O5—C13	−5.00 (19)	N2—C12—C14—O7	7.2 (4)
O1—Cu1—O7—C14	−106.32 (19)	N2—C12—C14—O8	−172.0 (2)
O3—Cu1—O7—C14	93.89 (19)	C11—C12—C14—O8	6.3 (4)
O5—Cu1—O7—C14	−4.4 (3)	C11—C12—C14—O7	−174.5 (3)
N1—Cu1—O7—C14	172.82 (19)	N3—C15—C20—C21	0.0 (4)
N2—Cu1—O7—C14	−2.56 (19)	C16—C15—C20—C19	−0.1 (4)
O1—Cu1—N1—C1	3.8 (2)	N3—C15—C16—C17	179.4 (3)
O1—Cu1—N1—C5	−177.5 (2)	C20—C15—C16—C17	−1.1 (5)
O3—Cu1—N1—C1	−178.8 (2)	C16—C15—C20—C21	−179.5 (3)
O3—Cu1—N1—C5	−0.2 (2)	N3—C15—C20—C19	179.5 (3)
O5—Cu1—N1—C1	−85.9 (2)	C15—C16—C17—C18	1.5 (5)
O5—Cu1—N1—C5	92.8 (2)	C16—C17—C18—C19	−0.6 (5)
O7—Cu1—N1—C1	95.4 (2)	C17—C18—C19—C20	−0.6 (5)
O7—Cu1—N1—C5	−85.9 (2)	C18—C19—C20—C15	0.9 (4)
O1—Cu1—N2—C8	−89.28 (19)	C18—C19—C20—C21	−179.7 (3)
O1—Cu1—N2—C12	98.1 (2)	C15—C20—C21—C22	−3.9 (4)
O3—Cu1—N2—C8	91.90 (19)	C19—C20—C21—N4	−2.1 (4)
O3—Cu1—N2—C12	−80.76 (19)	C19—C20—C21—C22	176.7 (3)
O5—Cu1—N2—C8	−1.46 (17)	C15—C20—C21—N4	177.3 (3)
O5—Cu1—N2—C12	−174.1 (2)	C20—C21—C22—C27	4.4 (4)
O7—Cu1—N2—C8	179.5 (2)	N4—C21—C22—C23	7.5 (4)
O7—Cu1—N2—C12	6.80 (18)	N4—C21—C22—C27	−176.8 (3)
Cu1—O1—C6—O2	−178.2 (2)	C20—C21—C22—C23	−171.4 (3)
Cu1—O1—C6—C1	3.0 (3)	C27—C22—C23—C24	2.1 (4)
Cu1—O3—C7—O4	−179.0 (2)	C21—C22—C27—N3	−0.9 (4)
Cu1—O3—C7—C5	0.8 (3)	C21—C22—C27—C26	−179.3 (3)
Cu1—O5—C13—O6	−171.3 (2)	C21—C22—C23—C24	177.9 (3)
Cu1—O5—C13—C8	9.7 (3)	C23—C22—C27—N3	175.1 (3)
Cu1—O7—C14—O8	177.6 (3)	C23—C22—C27—C26	−3.3 (4)
Cu1—O7—C14—C12	−1.6 (3)	C22—C23—C24—C25	0.3 (5)
Cu1—N1—C1—C2	177.5 (2)	C23—C24—C25—C26	−1.7 (5)
Cu1—N1—C1—C6	−3.3 (3)	C24—C25—C26—C27	0.5 (5)
C5—N1—C1—C2	−1.1 (4)	C25—C26—C27—N3	−176.3 (3)
C5—N1—C1—C6	178.1 (2)	C25—C26—C27—C22	2.1 (5)
Cu1—N1—C5—C4	−179.4 (2)	N5—C28—C29—C30	−179.7 (3)
Cu1—N1—C5—C7	0.6 (3)	C33—C28—C29—C30	1.3 (4)
C1—N1—C5—C4	−0.8 (4)	N5—C28—C33—C32	179.8 (2)
C1—N1—C5—C7	179.2 (2)	N5—C28—C33—C34	0.8 (4)
Cu1—N2—C8—C9	−171.50 (19)	C29—C28—C33—C32	−1.2 (4)
Cu1—N2—C8—C13	6.8 (3)	C29—C28—C33—C34	179.8 (3)
C12—N2—C8—C9	1.1 (4)	C28—C29—C30—C31	−0.1 (5)
C12—N2—C8—C13	179.4 (2)	C29—C30—C31—C32	−1.3 (5)
Cu1—N2—C12—C11	171.8 (2)	C30—C31—C32—C33	1.4 (5)
Cu1—N2—C12—C14	−9.9 (3)	C31—C32—C33—C28	−0.2 (4)
C8—N2—C12—C11	−1.0 (4)	C31—C32—C33—C34	178.8 (3)
C8—N2—C12—C14	177.3 (2)	C28—C33—C34—N6	177.9 (3)
C15—N3—C27—C26	175.2 (3)	C28—C33—C34—C35	−1.7 (4)
C15—N3—C27—C22	−3.2 (4)	C32—C33—C34—N6	−1.1 (5)
C27—N3—C15—C16	−176.8 (3)	C32—C33—C34—C35	179.3 (3)
C27—N3—C15—C20	3.7 (4)	N6—C34—C35—C36	−0.6 (5)
C28—N5—C40—C39	−179.1 (3)	N6—C34—C35—C40	−177.4 (3)
C28—N5—C40—C35	0.8 (4)	C33—C34—C35—C36	179.0 (3)
C40—N5—C28—C29	−179.3 (3)	C33—C34—C35—C40	2.2 (4)
C40—N5—C28—C33	−0.3 (4)	C34—C35—C36—C37	−177.3 (3)
C2—C1—C6—O1	179.1 (3)	C40—C35—C36—C37	−0.4 (5)
C2—C1—C6—O2	0.2 (5)	C34—C35—C40—N5	−1.7 (5)
N1—C1—C6—O2	−179.0 (3)	C34—C35—C40—C39	178.2 (3)
C6—C1—C2—C3	−177.3 (3)	C36—C35—C40—N5	−178.8 (3)
N1—C1—C6—O1	0.0 (4)	C36—C35—C40—C39	1.1 (5)
N1—C1—C2—C3	1.8 (4)	C35—C36—C37—C38	−0.3 (5)
C1—C2—C3—C4	−0.6 (4)	C36—C37—C38—C39	0.2 (5)
C2—C3—C4—C5	−1.2 (4)	C37—C38—C39—C40	0.6 (5)
C3—C4—C5—C7	−178.0 (3)	C38—C39—C40—N5	178.7 (3)
C3—C4—C5—N1	1.9 (4)	C38—C39—C40—C35	−1.2 (5)

### Hydrogen-bond geometry (Å, °)

**Table:**

*D*—H···*A*	*D*—H	H···*A*	*D*···*A*	*D*—H···*A*
N6—H40···O1W	0.93 (3)	2.04 (3)	2.927 (4)	159 (2)
O2W—H41···O6^i^	0.87 (4)	1.93 (4)	2.796 (3)	177 (4)
N4—H42···O4^ii^	0.86 (3)	1.97 (3)	2.808 (3)	165 (3)
N4—H43···O7^iii^	0.92 (4)	1.99 (4)	2.880 (3)	161 (3)
O3W—H44···O2W	0.86 (4)	1.86 (4)	2.720 (4)	176 (3)
N6—H45···O6	0.86 (3)	2.18 (3)	2.965 (3)	153 (3)
N5—H46···O8^iii^	0.82 (3)	1.91 (3)	2.719 (3)	173 (3)
O3W—H47···O4^iv^	0.84 (4)	1.95 (4)	2.780 (4)	168 (3)
O1W—H48···O5	0.87 (4)	1.97 (4)	2.828 (3)	174 (4)
N3—H49···O3W	0.84 (3)	1.86 (3)	2.698 (3)	170 (3)
O1W—H50···O2^v^	0.89 (5)	1.96 (5)	2.847 (4)	176 (4)
O2W—H51···O6	0.86 (6)	1.97 (5)	2.812 (4)	167 (4)
C3—H3···O2W^v^	0.94	2.57	3.266 (4)	131
C10—H10···O3^vi^	0.94	2.51	3.152 (3)	126
C19—H19···O4^ii^	0.94	2.49	3.401 (3)	163
C23—H23···O7^iii^	0.94	2.52	3.262 (3)	136

Symmetry codes: (i) −*x*+1, −*y*+1, −*z*+1; (ii) −*x*+1, −*y*, −*z*; (iii) *x*−1, *y*, *z*−1; (iv) *x*−1, *y*, *z*; (v) −*x*+1, −*y*, −*z*+1; (vi) −*x*+2, −*y*+1, −*z*+1.
